# Elastography in predicting preterm delivery in asymptomatic, low-risk women: a prospective observational study

**DOI:** 10.1186/1471-2393-14-238

**Published:** 2014-07-21

**Authors:** Slawomir Wozniak, Piotr Czuczwar, Piotr Szkodziak, Pawel Milart, Ewa Wozniakowska, Tomasz Paszkowski

**Affiliations:** 1 3rd Chair and Department of Gynecology, Medical University of Lublin, ul. Jaczewskiego 8, Lublin 20-090, Poland

**Keywords:** Elastography, Preterm delivery, Ultrasonography, Cervical assessment

## Abstract

**Background:**

Despite the efforts to decrease the rate of preterm birth, preterm delivery is still the main cause of neonatal morbidity and mortality. Identifying patients threatened with preterm delivery remains one of the main obstetric challenges. The aim of this study was to estimate the potential value of elastographic evaluation of internal cervical os stiffness at 18-22 weeks of pregnancy in low risk, asymptomatic women in the prediction of spontaneous preterm delivery.

**Methods:**

This prospective observational study included 333 low-risk, asymptomatic women presenting for the routine second trimester ultrasound scan according to the Polish Gynecological Society recommendation between 18-22 weeks of pregnancy. Ultrasound examinations of the cervix were performed transvaginally. The following data were recorded: elastographic color assessment of the internal os and ultrasound cervical length at 18-22 and 30 weeks of pregnancy; maternal age; obstetrical history; presence of cervical funneling at 30 weeks of pregnancy; gestational age at birth. Elastographic assessment of the internal os was performed using a color map: red (soft), yellow (medium soft), blue (medium hard) and purple (hard). If two colors were visible in the region of the internal os, the softer option was noted. Statistical analysis was performed using Statistica software (version 10, Statsoft Poland) using the following tests: chi square test to compare frequency of preterm deliveries in various categories of internal os assessment and Spearman correlation test to determine the correlation between elastographic assessment and cervical shortening. To determine the cut off category of internal os elastography assessment in selecting high preterm delivery risk patients we have calculated the sensivity, specifity, negative predictive value and positive predictive value.

**Results:**

The number of preterm deliveries (<37 weeks of pregnancy) was significantly higher in the red and yellow groups, than in the blue and purple groups. The sensivity, specifity, NPV and PPV for both red and yellow internal os assessment in predicting preterm delivery were 85.7%, 97.6%, 98.3% and 81.1% respectively.

**Conclusions:**

Elastographic assessment of the internal cervical os at 18-22 weeks of pregnancy may identify patients with high risk of preterm delivery in low-risk, asymptomatic women.

## Background

Preterm delivery is defined as premature birth before 37 completed weeks of gestation and is responsible for 75% of all neonatal deaths [1]. Despite the efforts to decrease the rate of preterm birth, the WHO estimated that 11.1% of all births were preterm [2]. The main obstetric challenge is to identify patients at high risk of preterm birth and prolong the pregnancy as long as possible: between the 23 and 32 week the mortality and severe handicap rates decrease dramatically [3-5].

Elastography is an ultrasound-based imaging technique visualizing the stiffness of examined region. The technique is based on the phenomenon, that after applying pressure with the probe, soft tissues are compressed in a greater extent than hard tissues and the gradient values of strain are visualized on a color map. Elastography found its use mainly in imaging of various tumors (such as breast, thyroid or prostate), where it provides important information on the tumor size, location, infiltration and optimal biopsy region [6-9]. An increasing number of reports shows the possible applications of elastography in obstetrics and gynecology, for instance in predicting the success of induction of labour [10,11] and differential diagnosis of endometrial pathologies [12].

It has been shown that collagenolytic activity in cervical tissue increases as the pregnancy progresses, and this process is more pronounced in case of cervical insufficiency, decreasing collagen content and modifying biomechanical properties of the cervix [13]. Moreover, such structural changes may be identified by elastography before the cervix begins to shorten [14]. In normal pregnancies the internal os and cervical canal are hard and allow the pregnancy to continue until term [15]. Swiatkowska-Freund and Preis investigated the relationship between the result of cervical elastography assessment and the success of induction of labor. The authors found, that it was only the stiffness of the internal os that predicted successful labor induction [11]. Hernandez-Andrade et al. reported a continuous reduction in cervical stiffness with decreasing cervical length and increasing gestational age, which manifested mainly in the internal cervical os [16]. It has also been suggested, that elastography may hold potential to predict risk of preterm birth [17].

The aim of this study was to estimate the potential value of elastographic evaluation of internal cervical os stiffness at 18-22 weeks of pregnancy in low risk, asymptomatic women in the prediction of spontaneous preterm delivery.

## Methods

**Figure 1:**
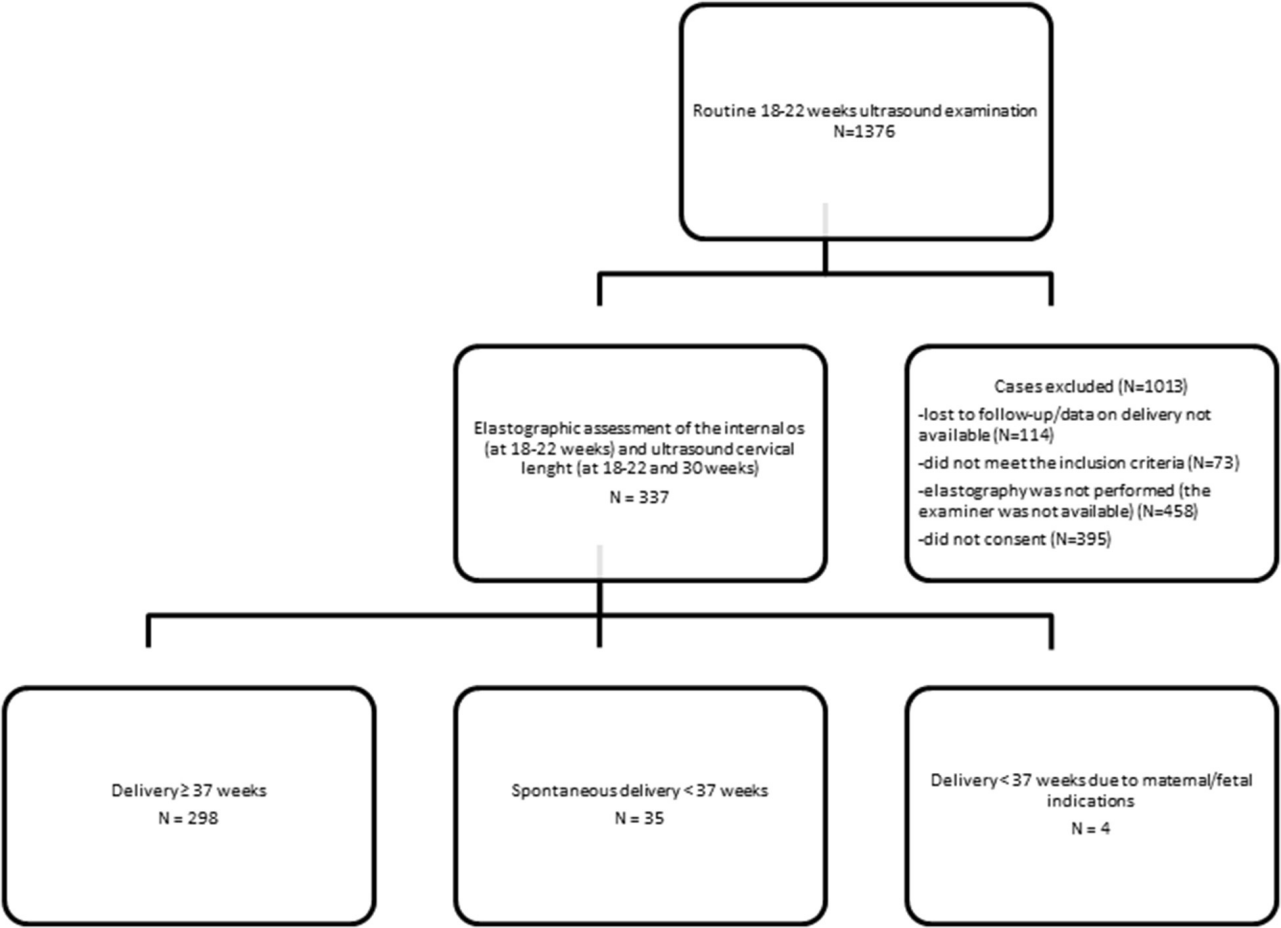
Flow chart of the study design.

This single institution prospective observational study included low-risk, asymptomatic women presenting for the routine second trimester ultrasound scan [18]. Inclusion criteria: normal, uncomplicated pregnancy, gestational age between 18 + 0 and 21 + 6 weeks; cervical length > 25 mm, singleton pregnancy, no uterine contractions, no evidence of preterm rupture of membranes, no cervical funneling, no cervical dilation, no preterm birth in history, no previous cervical surgery. The following data were recorded: elastographic color assessment of the internal os (at 18-22 weeks) and ultrasound cervical length (at 18-22 and 30 weeks); maternal age; obstetrical history; presence of cervical funneling at 30 weeks of pregnancy; gestational age at birth. In preterm deliveries the underlying cause was documented, cases due to maternal and fetal indications were excluded. Flowchart of the study design is shown in Figure 1. The study was approved by the Bioethical Committee of the Medical University of Lublin. Written informed consent was obtained from all participants. This study was prepared and performed according to the STROBE Statement Checklist for observational studies (Additional file 1).

**Figure 2:**
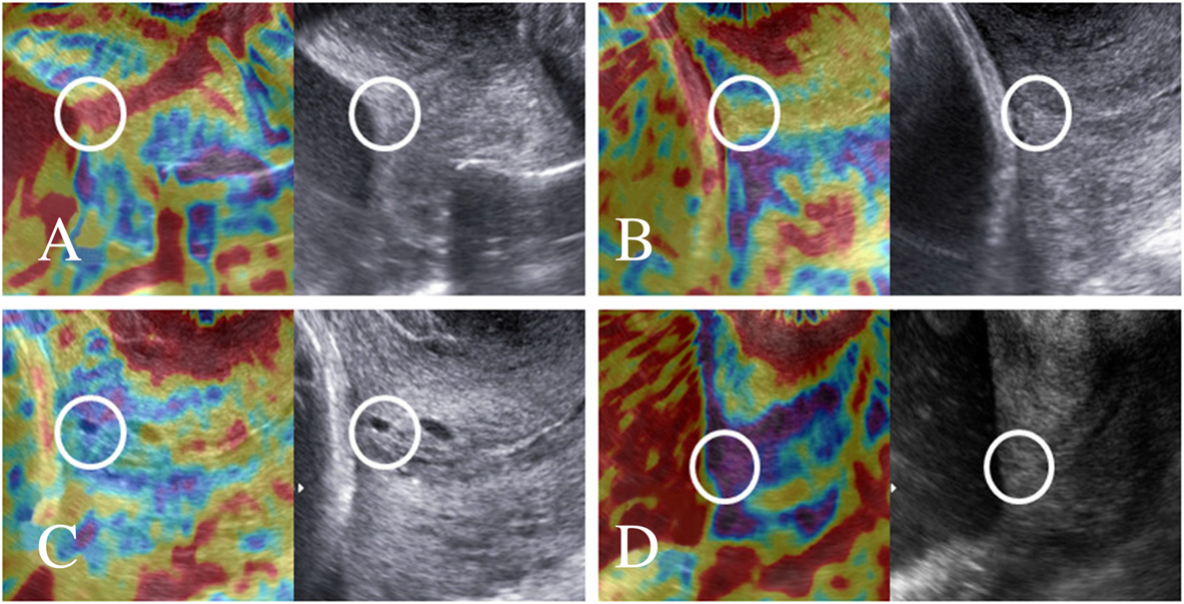
**Elastographic (left) and ultrasound (right) images of the internal cervical os (circles) at 18-22 weeks of pregnancy.** Internal cervical os stiffness was assessed as: **A)** soft (red); **B)** medium soft (yellow); **C)** medium hard (blue); **D)** hard (purple).

Ultrasound examinations of the cervix were performed transvaginally at 18-22 and 30 weeks of pregnancy by one experienced obstetrician (SW; 20 years of experience in ultrasound) on a Samsung Medison V20 Prestige equipped with a transvaginal (4 – 9 MHz) convex probe and Elastoscan® option. Cervical length measurements were performed as previously described [19]: in the sagittal plane with the entire cervical canal visible, calipers placed on the internal and external os, with an empty bladder and minimal pressure. Three measurements were performed, the shortest value was noted. The presence of funneling (defined as a protrusion of the amniotic membranes 3 mm or more into the internal cervical os [19] was also recorded. Elastography of the cervix was performed similarly to the technique reported by Swiatkowska-Freund and Preis [11]. The main differences in our study were that we have decided not to use the five step elastography index proposed by Swiatkowska-Freund and Preis and to evaluate the stiffness of the internal os only. The region of internal cervical os was identified subjectively as the cervical muscle surrounding the beginning of the cervical canal (Figure 2), excluding the mucus in the cervical canal. During the examination the patients were breathing normally and the operator did not apply pressure to the cervix – the elastographic image of the cervix was generated by patient’s breathing movements and arterial pulsation. After visualizing the sagittal section of the cervical canal elasticity of the internal os was assessed using a color map as: red (soft), yellow (medium soft), blue (medium hard) and purple (hard) (Figure 2). Any additional movements (patient changing position, coughing or even talking; the operator moving his hand) lead to the presence of artifacts and may change the appearance of the color map. Once a stable video sequence (at least 10 seconds) was obtained the operator decided about the dominant color of the internal cervical os, in case of difficulties (two colors of similar intensity visible) the softer option was noted.

Statistical analysis was performed using Statistica software (version 10, Statsoft Poland). The frequency of funneling at 30 weeks of gestation and preterm deliveries in various categories of internal os assessment were compared by the chi square test. To determine the cut off category of internal os elastography assessment in selecting high preterm delivery risk patients we have calculated the sensivity, specifity, negative predictive value (NPV), positive predictive value (PPV) and positive and negative likelihood ratios (LR + and LR-) with 95% confidence intervals (CI) for red (soft); and red and yellow (soft and medium soft, warm colors) and red, yellow and blue (soft and medium soft and medium hard). The correlation between elastographic assessment of internal os stiffness at 18-22 weeks of pregnancy and cervical length at 18-22 weeks and percentage cervical shortening between the 18-22 and 30 weeks were analyzed with the Spearman correlation test. P values < 0.05 were considered significant.

## Results

Between 2010 and 2013, 1376 patients presented for a routine 18-22 weeks ultrasound examination. Data about the delivery was obtained from 1262 patients, 76 (6.0%) of them delivered before 37 weeks of gestation. Three hundred and thirty seven patients met the inclusion criteria and agreed to participate in the study. Four patients had preterm deliveries due to maternal or fetal indications leaving 333 cases for analysis. All patients were of Caucasian racial origin. The clinical characteristics and obstetric history of the studied population are shown in Table 1.

**Table 1:** Clinical characteristics of the studied population

**Patient age (median, range)**			**27**	**18-42**
Obstetrical history	No of natural deliveries (n, %)	0	229	68.0
		1	99	29.4
		2	9	2.7
	No of cesarean sections (n, %)	0	289	85.8
		1	42	12.5
		2	6	1.8
	No of spontaneous abortions* (n, %)	0	249	73.9
		1	78	23.1
		2	10	3.0
Cervical length at 18-22 weeks of gestation (median, range)			44	27-57
Gestational age at delivery (median, range)			39.2	31.0-41.3

*All spontaneous abortions occurred before 12 weeks of pregnancy.

In the studied population 35 cases of spontaneous preterm birth (<37 weeks of pregnancy) occurred. At 18-22 weeks of pregnancy the internal os was evaluated as purple (hard) in 230, blue (medium hard) in 66, yellow (medium soft) in 10 and red (soft) in 27 patients. The numbers and percentages of preterm deliveries in respective groups were: purple – 3 (1.3%); blue – 2 (3.0%); yellow – 6 (60.0%); and red – 24 (88.9%). In the warm color group (red and yellow) preterm delivery occurred in 30 out of 37 patients (81.1%), whilst in the cold color group – in 5 out of 296 patients (1.7%). The number of preterm deliveries was not significantly different between purple and blue colors (cold colors) (p > 0.05) and between red and yellow colors (warm colors) (p > 0.05). The number of preterm deliveries in the red group was significantly higher than in the blue and purple groups (p < 0.001 and p < 0.001). The number of preterm deliveries in the yellow group was also significantly higher than in the blue and purple groups (p = 0.02 and p = 0.01).

The sensivity, specifity, negative predictive value (NPV), positive predictive value (PPV) and positive and negative likelihood ratios (LR + and LR-) with 95% confidence intervals for various categories of internal os elastography assessment in selecting high preterm delivery risk patients is shown in Table 2.

**Table 2:** Diagnostic accuracy of elastography performed at 18-22 weeks of pregnancy in predicting preterm delivery for various cut off colors: red (soft); yellow and red (soft and medium soft) and red, yellow and blue (soft, medium soft and medium hard)

	**Internal os elastography assessment**		
	**Red**	**Yellow and red**	**Blue, yellow and red**
Sensivity (95% CI)	68.6% (50.7-83.1)	85.7% (69.7-95.2)	91.4% (76.9-98.1)
Specifity (95% CI)	99.0% (97.1-96.4)	97.6% (95.2-99.0)	76.2% (70,9-80.9)
NPV (95% CI)	96.4% (93.6-98.2)	98.3% (96.1-99.4)	98.7% (96.2-99.7)
PPV (95% CI)	88.9% (70.8-97.6)	81.1% (64.8-92.0)	31.1% (22.3-40.9)
LR + (95% CI)	68.1 (21.6-214.7)	36.5 (17.3-76.8)	3.8 (3.0-4.8)
LR- (95% CI)	0.32 (0.19-0.52)	0.15 (0.06-0.33)	0.11 (0.04-0.33)

The Spearman correlation test showed a statistically significant, positive correlation between elastographic assessment of internal os stiffness and cervix length at 18-22 weeks of pregnancy (R = 0,3594; p < 0.001).

**Figure 3:**
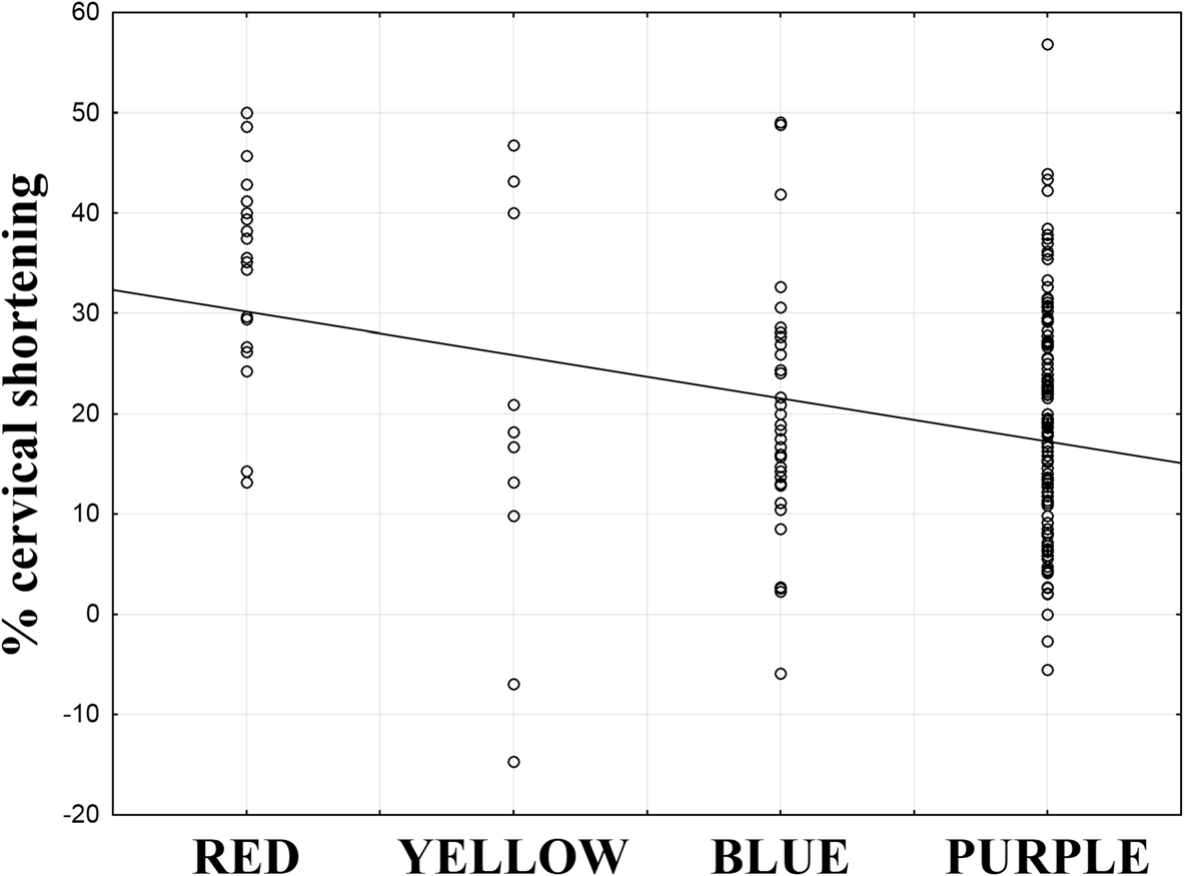
Correlation between elastographic assessment of internal os stiffness at 18-22 weeks of pregnancy and percentage cervical shortening between the 18-22 and 30 weeks scan (Spearman correlation test: R = -0.2; p < 0.001).

During the second examination at 30 weeks of pregnancy the median cervical length in the entire population was 36 mm (range 19 – 49). Median percentage cervical length decrease was 18% (range -14.7 – 56.9). The Spearman correlation test showed a statistically significant, negative correlation between elastographic assessment of internal os stiffness at 18-22 weeks of pregnancy and percentage cervical shortening between the 18-22 and 30 weeks scan (R = -0.2; p < 0.001) (Figure 3). The percentage of cases with cervical funneling at 30 weeks of pregnancy in various categories of elastographic internal os assessment is shown in Table 3. The risk of funneling was higher in the red group in comparison to the other groups.

**Table 3:** Percentage of cases with cervical funneling at 30 weeks of pregnancy depending on the category of internal os elastographic assessment at 18-22 weeks of pregnancy

**Internal os elastography assessment at 18-22 weeks**	**Number (%) of cases with funneling at 30 weeks**	**P value**			
		**Vs Red**	**Vs Yellow**	**Vs Blue**	**Vs Purple**
Red	17 (63.0%)	X	0.004	<0.001	<0.001
Yellow	1 (10.0%)	0.004	X	0.47	0.85
Blue	3 (4.5%)	<0.001	0.47	X	0.31
Purple	19 (8.3%)	<0.001	0.85	0.31	X

## Discussion

The principal findings of this study are that, firstly, elastography allows the assessment of internal cervical os stiffness in pregnancy. Secondly, elastography may identify patients with high risk of preterm birth in the low-risk, asymptomatic population. Finally, elastographic abnormalities may precede and predict clinical and ultrasound findings (cervical shortening and funneling).

At present screening for patients with high risk of preterm birth is based mainly on the measurement of cervical length at the second trimester scan [20-22]. However, it had been shown, that when using the cut-off value of 25 mm, measurement of cervical length at 24 weeks of pregnancy had a sensivity of 37.3% in predicting delivery before 35 weeks [19]. This means, that out of all cases of preterm deliveries (< 35 weeks) in 62.7% the cervix length was above 25 mm. These findings justify the search for additional diagnostic tests in patients with normal cervix length. Our results show, that even in asymptomatic patients with a normal cervix length at 18-22 weeks scan it is possible to identify a group of patients with high risk of preterm birth. It has to be stressed that all of these patients according to current guidelines [18] were classified as low-risk of preterm birth and did not qualify for additional interventions and monitoring. Elastographic assessment of the internal cervical os may provide additional information in preterm delivery risk assessment. On the other hand, it has to be stressed that the pathogenesis of preterm delivery is multifactorial, and the screening cannot be based only on the assessment of the cervix, even though cervix length is one of strongest predictors of preterm delivery [1]. For the purpose of this study we decided to focus on a selected population of low-risk patients, with the inclusion criteria designed to exclude the majority of other causes of preterm deliveries.

It is known that the properties of cervical tissue change during pregnancy and labor. Mechanical devices have been used to objectively assess cervical mechanical properties in pregnancy: it has been shown that the cervix becomes softer [23] and more distensible [24] as the pregnancy progresses. It has to be noted, that the cervix undergoes a variety of changes during pregnancy. Two terms have to be distinguished: cervical softening and cervical ripening. Cervical softening refers to changes in tissue properties and composition caused by the process of remodeling of the extracellular matrix; occurs gradually during pregnancy and precedes effacement and dilation [25]. On the other hand, ripening is connected with leukocyte migration and release of proinflammatory cytokines into the cervical stroma; results in loss of structural function of the cervix and manifests clinically by an elevated Bishop score (i.e. shortening, effacement and dilation) [25]. Hernandez-Andrade et al. demonstrated that cervical tissue strain assessed by elastography was more strongly associated with cervical length than with gestational age at examination [16]. That is why elastography seemed a perfect tool to search for patients with increased risk of preterm birth: it may show softening of the cervix, which appears prior to shortening and funneling that are identified by ultrasound. Moreover, the internal os (that was evaluated in our study) is not available for palpation.

Previous groups investigating elastographic properties of the cervix defined fixed dimensions of their regions of interest (10 mm [14] or 6 mm [26]), but it has been suggested that such approach is not beneficial for standardization of the procedure [27] as the dimensions of the cervix vary between women [28].

Our results show, that patients, whose internal os was evaluated as soft at the 18-22 weeks scan had a significantly higher risk of funneling and significantly higher percentage cervical shortening at 30 weeks, therefore we conclude, that elastographic abnormalities precede ultrasound and clinical findings.

Fruscalzo and Schmitz suggested that the major weakness of cervical elastography is the lack of standardization of the force applied [29]. At present various groups propose different standardization approaches. For instance, Molina et al. advanced the probe into cervical tissue by about 1 cm [26], Fruscalzo et al. compressed the cervix to obtain maximal compression of the anterior lip, until the posterior lip was displaced [30] while Swiatkowska-Freund generated elastographic images by patient’s breathing and arterial pulsation without any additional force applied [11]. We have used the last of these techniques – it has to be stressed that since no reliability studies of Swiatkowska-Freund’s method were performed, it seems of utmost importance to investigate the operator-independence and the reproducibility of this technique.

It has been suggested that the elastographic color of the cervix is not homogenous [31]. Preis et al. found, that the hardest part of the cervix is the middle part of the posterior lip [10]. Molina et al. reported similar results, with the hardest region described as internal inferior cervical lip. The authors hypothesized, that this may be due to the fact, that these regions are further away from the probe and therefore the pressure applied and the compression of the tissues is less, than in the parts closer to the probe [26]. The authors investigated both the intraobserver and interobserver reproducibility of elastrographic assessment of the cervix and obtained reliable, reproducible measurements, except for the external superior part of the cervix evaluated by two different observers [26]. This is the part of the cervix compressed by the probe, and probably the interobserver discordance was caused by different force applied by the examiners. Interestingly, evaluation of other parts of the cervix remained independent of interobserver variability, despite the abovementioned possible differences in the examination technique. Similarly, Hernandez Andrade et al. observed that interobserver agreement was highest in the internal os region [16]. Therefore it can be assumed, that elastographic examination of the internal os is in a lesser extent affected by intra- and interobserver bias. Moreover, in our study we have used the examination technique described by Swiatkowska-Freund and Preis based on Elastoscan® software, which does not require applying additional pressure to the cervix and may further decrease the interobserver variability [11].

Our study has some limitations. First of all, as discussed above, elastography lacks standardization. That is why to avoid the possible interobserver bias only one experienced examiner performed all examinations. This of course may question the generalizability of the results of this study. However, our study is an initial observation, showing that by using elastography it is possible to identify patients threatened with preterm delivery in the low-risk population. Naturally, before even suggesting the inclusion of elastography into the routine second trimester ultrasound examination further studies focusing mainly on the standardization and intra- and interobserver variability of this technique are needed. The percentage of preterm delivery in our study group was surprisingly high (11.6%). The only explanation is that the number of patients who had elastography performed was too small to assess the general risk of preterm delivery. Including all the patients for whom the data on delivery was available decreased the preterm delivery rate to 6.0%. Finally, only a small percentage (24.5%) of patients presenting to our department for the second trimester scan were included in the study. Many patients did not agree to participate in the study, but more importantly in many cases the examiner (SW) was not available during the examination and due to the study design (all examinations performed by one person) elastography was not performed.

In our study we have assessed the stiffness internal cervical os using a four step color scale. Two categories were used to describe softer internal cervical orifices: red (soft) and yellow (medium soft). After comparing the diagnostic accuracy of elastography in predicting the risk of preterm birth for only red or red and yellow internal os evaluation, we have observed an increase in sensivity (84.6 vs 66.7% respectively), comparable specifity and NPV, and a small decrease of PPV (82.5 vs 89.7% respectively). Including the blue color in the high risk group resulted in a further increase of sensivity to 91.4%, but also increased the false positive rate to 23.8%. Therefore we postulate to include both warm colors (red and yellow) as predictors of high risk of preterm delivery.

## Conclusions

1. Elastographic assessment of the internal cervical os at 18-22 weeks of pregnancy may identify patients with high risk of preterm delivery in the low-risk, asymptomatic population.

2. Elastographic abnormalities may precede ultrasound and clinical findings, such as cervical shortening and funneling.

## Competing interests

The authors declare that they have no competing interests.

## Authors’ contributions

SW – designed the study, drafted the manuscript, searched the literature, performed the statistical analysis, performed ultrasound examinations. PC – contributed to the study design, drafted the manuscript, searched the literature, performed the statistical analysis and prepared the figures. PS – collected data, prepared the figures, participated in the study design. PM – revised the article, coordinated the study, performed the statistical analysis. EW – collected data, searched the literature, drafted the article. TP – participated in the study design, coordinated the study and supervised the research group, revised the article. All authors read and approved the final manuscript.

## Pre-publication history

The pre-publication history for this paper can be accessed here:

http://www.biomedcentral.com/1471-2393/14/238/prepub

## Supplementary Material

Additional file 1 STROBE Statement—checklist of items that should be included in reports of observational studies. Click here for file
